# Supplementary material for: Asynchronous magnetic resonance elastography: Shear wave speed reconstruction using noise correlation of incoherent waves
Source: Magn Reson Med. 2022 Oct 27;89(3):990–1001. doi: 10.1002/mrm.29502 (PMC9792433; doi:10.1002/mrm.29502)
Supplement: Supplementary file 1 — DATA S1 MATLAB code used to generate the numerical simulations of Figure 1 [file MRM-89-990-s002.zip › k-Wave/helpfiles/checkStability.html]

checkStability :: Functions (k-Wave)


# checkStability

Compute maximum stable time step for k-space fluid models.

## Syntax

```
dt_stability_limit = checkStability(kgrid, medium)
```

## Description

`checkStability` calculates the maximum time step for which the k-space propagation models `kspaceFirstOrder1D`, `kspaceFirstOrder2D` and `kspaceFirstOrder3D` are stable. These models are unconditionally stable when the reference sound speed is equal to or greater than the maximum sound speed in the medium and there is no absorption. However, when the reference sound speed is less than the maximum sound speed the model is only stable for sufficiently small time steps. The criterion is more stringent (the time step is smaller) in the absorbing case. The time steps given are accurate when the medium properties are homogeneous. For a heterogeneous media they give a useful, but not exact, estimate.

## Inputs

|  |  |
| --- | --- |
| `kgrid` | k-Wave grid object returned by `kWaveGrid` |
| `medium` | Structure containing the medium properties |

## Outputs

|  |  |
| --- | --- |
| `dt_stability_limit` | The maximum time step for which the models are stable. This is set to `Inf` when the model is unconditionally stable. |

## See Also

`kWaveGrid`, `kspaceFirstOrder1D`, `kspaceFirstOrder2D`, `kspaceFirstOrder3D`
